# Oleic Acid Metabolism in Response to Glucose in *C. elegans*

**DOI:** 10.3390/metabo13121185

**Published:** 2023-12-06

**Authors:** Andre F. C. Vieira, Mark A. Xatse, Sofi Y. Murray, Carissa Perez Olsen

**Affiliations:** Department of Chemistry and Biochemistry, Worcester Polytechnic Institute, Worcester, MA 01609, USA; afvieira@wpi.edu (A.F.C.V.); maxatse@wpi.edu (M.A.X.);

**Keywords:** oleic acid, glucose, phospholipids, mass spectrometry, stable isotopes, lipidomics, fatty acid metabolism

## Abstract

A key response to glucose stress is an increased production of unsaturated fatty acids to balance the increase in saturated fatty acids in the membrane. The *C. elegans* homolog of stearoyl-CoA desaturase, FAT-7, introduces the first double bond into saturated C18 fatty acids yielding oleic acid, and is a critical regulatory point for surviving cold and glucose stress. Here, we incorporated ^13^C stable isotopes into the diet of nematodes and quantified the ^13^C-labelled fatty acid using GC-MS and HPLC/MS-MS to track its metabolic response to various concentrations of glucose. Previous work has analyzed the membrane composition of *C. elegans* when responding to mild glucose stress and showed few alterations in the overall fatty acid composition in the membrane. Here, in nematodes exposed to higher concentrations of glucose, a specific reduction in oleic acid and linoleic acid was observed. Using time courses and stable isotope tracing, the response of fatty acid metabolism to increasing levels of glucose stress is characterized, revealing the funneling of monounsaturated fatty acids to preserve the abundance of polyunsaturated fatty acids. Taken together, higher levels of glucose unveil a specific reduction in oleic and linolenic acid in the metabolic rewiring required to survive glucose stress.

## 1. Introduction

As animals encounter perturbation in their environment or their diet, there is often a rewiring of metabolic pathways that tunes or adjusts membrane composition to new conditions [1,2]. In certain circumstances, like temperature changes, a different phospholipid content is established that promotes membrane function with the altered requirements of the membrane [1,3]. There are also situations, such as with the addition of moderate glucose stress to the diet, where the membrane composition remains stable; however, this stability requires a shift in the metabolic pathways to alter the flux of certain lipid species to the membrane. The PAQR-2 response network positively regulates enzymes, including FAT-7, which is essential in the production of unsaturated fatty acids (UFAs), and ELO-5, which is responsible for the synthesis of monomethyl-branched chain fatty acids (mmBCFAs). PAQR-2 is a membrane sensor that is homologous to the mammalian protein AdipoR2, which plays a similar role in regulating membrane fluidity in the membrane of HEK 293 cells [4,5,6]. The upregulation of both FAT-7 and ELO-5 is needed for the survival of glucose stress, highlighting the importance of fatty acid metabolism in this response [7,8,9,10]. 

To promote the correct balance of saturated fatty acids (SFAs) and unsaturated fatty acids (UFAs), fatty acids incorporated from the diet or produced by de novo synthesis must be processed and incorporated into the membrane in the correct ratios [11,12]. In *Caenorhabditis elegans* (*C. elegans*), FAT-7 introduces the first double bond to stearic acid (C18:0), producing oleic acid (C18:1n9), which is further elongated and desaturated to produce PUFAs (polyunsaturated fatty acids) including linoleic acid (C18:2n6) and the C20 PUFAs including eicosapentaenoic acid (C20:5n3) [13]. Here, in addition to their common names used for simplicity, we use a nomenclature of CX:YnZ, where X stands for the number of carbons, Y indicates the number of double bonds, and Z defines the location of the first double bond. It is important to note that the FA composition of *E. coli* (OP50), the standard laboratory diet used to feed *C. elegans* nematodes, consists primarily of saturated FAs and cyclopropyl FAs [7]. Therefore, to produce and incorporate polyunsaturated fatty acids (PUFAS) in the membrane, FAT-7 is essential for the conversion of C18:0 to C18:1n9 and, thus, the production of PUFAs in the nematode [9,14]. Additionally, the quantification of new fatty acid incorporation in *C. elegans* shows that the overall lipid production is compromised after FAT-7 RNAi knockdown, implicating this enzymatic step in overall fatty acid metabolism 12. Furthermore, fat-7 is upregulated upon cold and glucose exposure [7,15]. Together, both findings reinforce the importance of the conversion from C18:0 to C18:1n9 in membrane homeostasis. 

To promote membrane adaptation, mechanisms that adjust the production and allocation of different lipid molecules must be present. Techniques using ^13^C or ^15^N stable isotopes have been used to evaluate and quantify the dynamics of lipids in the phospholipid (PL) membrane. The evaluation of ^13^C stable isotope labeling in FAs showed that membrane rejuvenation is rapid in nematodes, with most membrane lipids being replaced within 24 h in young adults [11,12]. Recently, stable isotope studies showed that although the overall composition of the membrane is nearly unchanged with mild (15 mM) glucose stress, there are significant changes to the dynamics of fatty acids like C16:0 and 15-methyl hexadecanoic acid (C17iso) [7]. These altered labeling patterns led to the investigation of the role of monomethyl-branched chain fatty acids in glucose response, where it was shown that the production of these fatty acids is essential to survival in glucose, highlighting the utility of probing dynamics along with the overall abundance of fatty acids. 

Glucose supplementation in the diet reduces the lifespan of nematodes and reduces their resistance to stress. Research has also shown that a gradual elevation in glucose stress has an inversely proportional effect on the nematode’s survival [16,17]. The incorporation of glucose was previously shown to alter the overall FA composition in the bacteria, increasing the levels of C16:0 8. We recently found that *E. coli* growing in high-growth (HG) media enriched with glucose had lower levels of vaccenic acid (C18:1n7), suggesting its direct influence on the membrane composition assessed in nematodes [7]. Moreover, fat-7 expression in nematodes was shown to induce a dose-dependent response to increased levels of glucose stress [18]. The content of the diet also impacts the regulation and adaptation of membrane metabolism pathways [19]. Many fatty acids present in nematodes are partially obtained intact from the diet; therefore, their modification in bacterial composition also influences phospholipid metabolism in nematodes. The role of bacterial metabolism are not fully understood in the processing of glucose, but studies have found that living bacteria is necessary to see the longevity and healthspan effects on the nematode [20]. 

Much of what we know about the response to glucose is from studies that compromise the response to glucose via a reduction in PAQR-2, FAT-7, or ELO-5. Because of the relationship between glucose stress and membrane adaptation, we hypothesize that higher levels of glucose could reveal other mechanisms not seen previously in wildtype nematodes. Here, we assessed the membrane response to higher concentrations of glucose using GC-MS and HPLC-MS/MS to completely evaluate the response. In performing this task, we revealed specific alterations in oleic acid metabolism and could identify the requirement for a recovery period to correct the membrane composition. 

## 2. Materials and Methods

### 2.1. Maintenance of Strains and Population Synchronization

All experiments were conducted using wildtype N2 nematodes obtained from the *C. elegans* Genetics Center (CGC, Minneapolis, MN, USA). To synchronize each population of animals, gravid adults were treated with a 20% bleach solution, and the recovered eggs were washed at least 3 times with approximately 10 mL of M9 (a buffered salt solution). After each wash, the eggs were centrifuged, and the supernatant was discarded. The resulting eggs were left rotating overnight at 20 °C in an M9 solution. OP50 bacteria on HG plates were used in this study unless otherwise stated. To prepare bacterial stocks, 5 mL of LB cultures was inoculated with 2–3 colonies from an OP50 stock plate and left shaking at 37 °C overnight. In total, 100 uL from starter cultures were used to seed an HG (“high growth”) plate and left overnight at 20 °C before the nematodes were plated. All nematodes were kept at 20 °C for the duration of the experiment. Unless otherwise stated, 5000 animals were plated for each condition.

### 2.2. Stress Conditions, Labeling Strategy, and Time-Course Experiments

The preparation of glucose stress plates (+gluc) followed the protocol that we published previously [7]. Briefly, the +gluc plates were made to a final concentration of 15 mM, 100 mM, or 200 mM of glucose by adding a filtered glucose solution to cooled autoclaved HG media. All plates were seeded using regular OP50 bacteria, and the +gluc plates were seeded at least 4 days before plating the worms. To start the stress, synchronized L4 stage worms growing on HG plates were transferred to +gluc plates and fed for 12, 24, 48, and 72 h. 

Our labeling strategy involves feeding the nematodes a mixture of isotopically labeled food and unlabeled food as previously established [11]. In addition to monitoring the incorporation of dietary carbon (marked by the ^13^C isotopes), this mixture allows for the determination of synthesized fatty acids as fatty acids absorbed from the diet can be either fully ^12^C or fully ^13^C while de novo synthesized fatty acids are a mixture of ^12^C and ^13^C acquired from the digested bacteria. Briefly, Isogro media (^13^C) and LB media (^12^C) were inoculated with OP50 colonies to allow the growth of bacteria for 16 h at 37 °C. Next, the bacteria were harvested and resuspended in M9 at a concentration of 0.15 g/mL. This concentration was previously determined to allow the nematodes to access adequate food for the duration of the experiment [11]. A mixture containing enriched bacteria ^13^C:^12^C (60%:40%) was transferred to agarose plates and allowed to dry. Nematodes from +gluc plates were collected, washed three times using M9, and plated onto stable isotope labeling plates containing 800 uL of the bacteria mixture for 6 h. Labeled worms were removed from the labeling plates, washed, and stored at −80 °C until lipid extraction and analysis via mass spectrometry. 

Labeling with stable isotopes (^13^C) allows for the analysis of the percentage of all newly incorporated FA species simultaneously. Briefly, to calculate the amount of new fatty acids found in the nematodes, the isotopomers were normalized to the m+0 isotopomer and corrected for the incorporation of natural isotopes. The % of newly incorporated fatty acids considers all newly modified fat independent of its source (de novo synthesized, elongated, or directly absorbed), as described by Dancy et al., 2015 [12]. Error bars of the ^13^C labeling show the standard error of the mean, and t-tests were used to identify significant differences between the fatty acids. The t-tests were calculated using the GraphPad Prism software version 9.4.1 from 18 July 2022. Samples were considered significantly different if *p* < 0.05 when running an unpaired t-test and using the F-test to compare variances. 

### 2.3. Conditions with Recovery Period after Glucose Stress 

The glucose plates (+gluc) were made, as described above, to a final concentration of 100 mM of glucose. Synchronized L4 stage worms were transferred to +gluc plates and followed the stress as described. For 12 h (no recovery), approximately 5000 nematodes spent 12 h on glucose plates and were collected and washed three times before immediately snap-freezing for further GC/MS analysis. For 12 h (recovery), nematodes spent 12 h on glucose plates and 6 h of the “recovery period” on agarose plates seeded with concentrated OP50 (0.15 mg/mL) to mimic the conditions of stable isotope-labeling plates. After recovery, animals were collected and frozen for further analysis using GC-MS. Nematodes (no recovery) spent 18 h on glucose plates and were collected and washed three times before immediately snap-freezing for further GC/MS analysis. Statistical analysis used GraphPad Prism software and samples were considered significantly different if *p* < 0.05, if they were marked by the * sign if *p* < 0.1, or if they were marked by the # sign. 

### 2.4. Lifespan Analysis 

To quantify survival on +gluc plates, L4440 bacteria were seeded onto 3 cm NGM + CI plates, and synchronized L1 worms from bleached gravid adults were grown for 48 h, as previously described [7,20]. Approximately 50 L4-stage nematodes were then transferred to fresh control plates (NGM + CI) or fresh treatment plates (NGM + CI + gluc) and kept at 20 °C for the duration of the experiment. Each day, the number of dead animals was determined by gently prodding with a pick. Any nematodes that were lost due to crawling off the plate or via bagging were not counted in the analysis. 

### 2.5. Heat-Killed Bacteria Tests 

Concentrated OP50 *E. coli* stocks were generated from OP50 stock plates via inoculating 2–3 colonies in 4 flasks of 50 mL of LB media. These flasks were left to shake overnight (16–18 h) at 37 °C. Bacteria were pelleted from each flask and resuspended in M9 according to the pellet’s mass at a concentration of 0.15 g/mL. Two of the four resuspended pellets were heat-killed (HK) at 65 °C for 20 min. All four tubes of living and heat-killed bacteria were centrifuged at 3900 rpm for 10 min to re-pellet. In order to test the importance of living bacteria in processing glucose, glucose was added to the heat-killed bacteria and referred to as OP50 HK + gluc. The volume of media in each HG plate was considered 30 mL, and glucose was added to 100 mM for the +gluc conditions. The two remaining tubes of living and dead bacteria pellets were resuspended in the M9 buffer (OP50 and OP50 HK, respectively). 

The heat-killed bacteria were streaked on LB plates and left overnight in a 37 °C incubator to confirm death. All tubes were kept at room temperature for about 12–14 h until they were seeded. Before the plates were seeded, the tubes holding heat-killed bacteria were placed again in a 65 °C water bath for 20 min to ensure no survival among the bacteria. Nematodes in the L4 stage were transferred to each plate condition (OP50, OP50 + gluc, OP50 HK, and OP50 HK + gluc) right after plates were completely dry and allowed to feed for 12 h before they were frozen and saved for analysis. 

### 2.6. Lipid Extraction and GC Analysis 

The total lipid was extracted using a chloroform/methanol solution (2:1 mixture), and the PL population was separated using chromatography before GC-MS analysis [11,12]. Lipid standards, 1,2-diundecanoyl-sn-glycero-3-phosphocholine (Avanti Polar Lipids, Alabaster, AL, USA) and tritridecanoin (Nu-Chek Prep, Elysian, MN, USA), were added to each sample and used as an internal control to confirm the separation of lipid classes. Dried total lipids resuspended in 1 mL of chloroform were loaded onto HyperSep Silica SPE columns (100 mg capacity, Thermo Scientific, Waltham, MA, USA), and after a sequence of chloroform (3 × 1 mL), acetone/methanol (9:1; 5 × 1 mL) and methanol (3 × 1 mL) was used for each lipid class (i.e., phospholipids (PLs), glycolipids (GLs), and neutral lipids (NLs)) from the column. 

In this study, the focus was on the fatty acids associated with purified PLs; thus, the PL fraction was dried and resuspended in 1 mL of 2.5% H_2_SO_4_ in methanol before being incubated for 1 h at 80 °C to create fatty acid methyl esters (FAMEs) from the PL fraction. To recover the FAMEs, 200 µL of hexane was added, and the sample was mixed vigorously. The aqueous phase was frozen in dry ice, and an ethanol bath to ensure the easy recovery of the FAMEs in hexane, and 1–2 µL of FAMEs was analyzed using GC-MS (Thermo Trace 1310 GC with an ISQ LT single quadrupole mass spectrometer). The GC was fitted with a (5%)-phenyl)-methylpolysiloxane phase capillary column (HP-5ms, Agilent, Santa Clara, CA, USA). 

The relative % of FAs in each sample was calculated by using the integrated area under the peaks seen in the gas chromatograph and was quantified using the Thermo Fisher software Chromeleon version 7.2.10 ES. The error bars of the lipid composition show the standard error of the mean, and *t*-tests were used to find significant differences between the fatty acids. The *t*-tests were calculated using the GraphPad Prism software version 9.4.1 from 18 July 2022. Samples were considered significantly different if *p* < 0.05 when running an unpaired *t*-test while also using the F-test to compare variances. 

### 2.7. Quantification the Synthesis of Fatty Acids 

To determine the percentage of synthesized fatty acids, we followed the published protocol [11,12]. Briefly, using the variation in mass in the parent ion (m + 1, m + 2, etc.) analyzed using GC-MS, we excluded peaks directly incorporated from the diet and elongated from dietary content. More specifically, the molecular weight of C16:0 can vary from 270 to 286 depending on the number of stable isotopes (^13^C) that are incorporated into the molecule. Based on the examination of bacterial data and the normalization of the natural incorporation of ^13^C, peaks between 270 and 272 and 284 and 286 were considered directly incorporated or elongated from the diet. Therefore, to calculate the synthesis of FA, peaks between 273 and 283, after normalization, were selected. The percentage calculation takes into consideration the sum of 273–283 peaks over the sum of total peaks 270–286 multiplied by 100%, as represented in the following equation: synthesis % of FA = (Σ273-283)/(Σ270-286) × 100.

### 2.8. Phospholipid Extraction and Analysis Using HPLC-MS/MS 

The phospholipid analysis was conducted based on previous studies [12]. Briefly, the total phospholipids were extracted from frozen nematodes based on the Folch procedure using 2:1 chloroform/ methanol. Extracted lipids were resuspended in 200 μL of acetonitrile/2-propanol/water (65:30:5 *v*/*v*/*v*), and 10 μL was injected into the Dionex UHPLC UltiMate 3000. The phospholipids were separated on a reverse-phase LC column (C18 Hypersil Gold 2.1 × 50 mm, 1.9 μm column) at a flow rate of 300 µL/min. The phospholipids were eluted using gradient solvents A and B containing 10 mM ammonium formate (NH_4_COOH) and 0.1% formic acid (FA). Solvent A was composed of 60/40 water/acetonitrile, and solvent B was composed of 90/10 isopropyl alcohol/acetonitrile. The schedule for the gradient was 32% B over 0–1.5 min; 32–45% B from 1.5 to 4 min; 45–52% B from 4 to 5 min; 52–58% B from 5 to 8 min; 58–66% B from 8 to 11 min; 66–70% B from 11 to 14 min; 70–75% B from 14 to 18 min; 75–97% B from 18 to 21 min; 97% B up to 25 min; 97–32% B from 25 to 26 min; 32% B was maintained for 30 min for column equilibration. 

The mass spectrometry of PLs was performed on a Q Exactive mass spectrometer (Thermo Fisher Scientific, Waltham, MA, USA). The analysis was performed in the negative ion mode and the full scan data-dependent MS2 (ddMS2) mode. The scanning range for MS analysis was 300–1200 *m*/*z* with a maximum injection time of 100 ms and the AGC target 10^6^. The capillary spray voltage was set at 3.2 kV, and the capillary temperature was set at 325 °C. The sheath gas flow rate was at 45 units, and the auxiliary gas flow was set at 10 units. For MS1 profiling, scans were run at a resolution of 70 k. MS2 analyses were performed at an NCE of 35 using 6 scan events, with the top 5 ions chosen from an initial MS1 scan. 

An analysis of the LC-MS/MS data was conducted using the software Lipid Data Analyzer (LDA) Version 2.8.1. A 0.1% relative peak cutoff value was applied to the RAW files to focus on the major phospholipid species [21].

## 3. Results

### 3.1. High Dietary Glucose Alters the Allocation of Oleic Acid to the Membrane 

**Figure 1:**
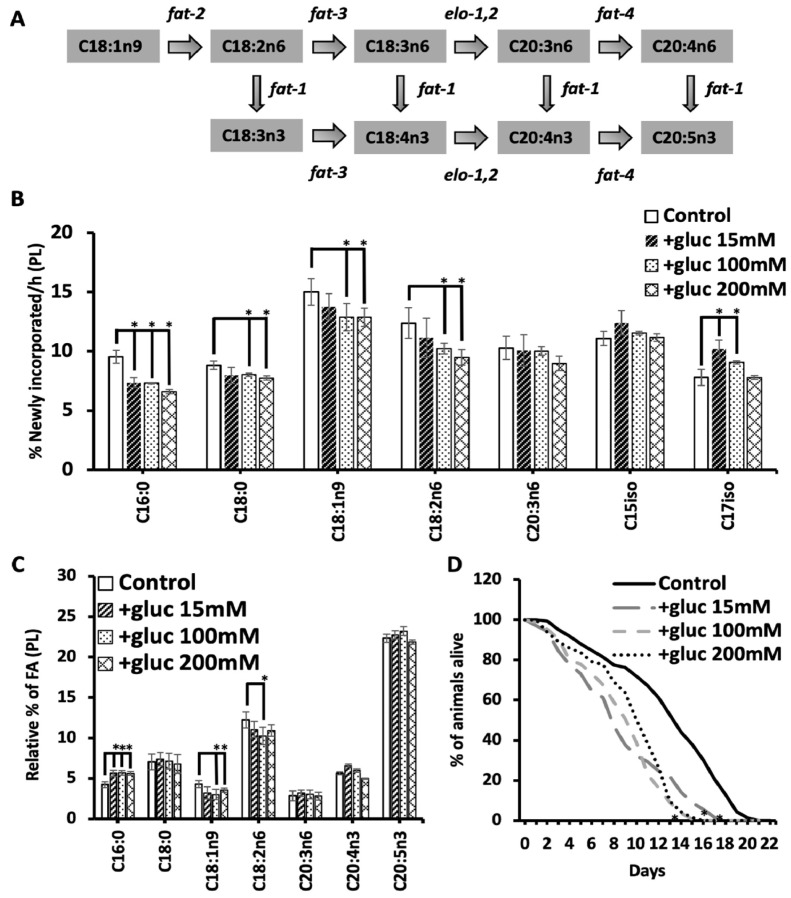
High concentrations of glucose reveal a compromised flux of oleic and linoleic acids. (**A**) The fate of oleic acid (C18:1n9) in the elongase and desaturase pathway is shown by the desaturases (“fat” genes) listed. (**B**) L1 nematodes were grown for 48 h, followed by 12 h of glucose stress at 15 mM (black dashed), 100 mM (black dots), and 200 mM (black hatch). After glucose treatment, nematodes were labeled by feeding^13^C:^12^C (60:40) for 6 h. Multiple fatty acids species showed significant differences in the % of newly incorporated fatty acids when compared to the control populations (white bars). (**C**) The relative % of the fatty acids upstream and downstream of oleic acid was determined in the same populations of animals described above. (**D**) Lifespans showed a significantly shorter mean and maximum lifespan in animals fed glucose at all concentrations at L4. All GC/MS analysis values represent the mean ± SEM of at least 9 biological replicates. Statistical significance (*p* < 0.05) was calculated for each condition compared to the control and is indicated by * and was calculated using unpaired *t*-tests and F-tests to compare variances. Survival curves are presented as means of at least three independent replicates with *n* = 50 nematodes/conditions. Statistical analysis for lifespan analysis was performed via the Log-rank (Mantel-Cox) test. *p* ≥ 0.05 is represented by *.

In nematodes supplemented with low levels of glucose (15 mM), the dynamics of specific fatty acids are altered as they are assayed via stable isotope labeling; however, most fatty acid species do not show significant changes [7]. To further probe the membrane adaptations needed with glucose supplementation, we fed nematodes increasing concentrations of glucose (referred to as +gluc) from 15 mM (mild stress) to 200 mM (high stress) and profiled the dynamics of the fatty acid populations via stable isotope feeding and GC-MS (Figure 1B). Fatty acids were considered new to the membrane if the molecular weight of intact FA was increased by at least one mass unit (MW + 1, MW + 2, MW + 3, etc.) after correction for the natural abundance of stable isotopes in the environment and the presence of ^12^C in the labeled OP50 (Isogro media, Sigma Aldrich, St. Louis, MO, USA).

At the lowest concentration (15 mM), there was a significant reduction in ^13^C incorporation into C16:0 and a significant increase in the ^13^C incorporation in C17iso, consistent with our previous reports [7]. As the amount of glucose increased, the levels of newly incorporated C16:0 were reduced from 9.5% ± 0.5 in the controls to 7.3% ± 0.1 and 6.6% ± 0.05 in 100 mM and 200 mM, respectively (Figure 1B). There was no further decrease in C16:0 abundance with increasing glucose concentrations. Interestingly, the increase in C17iso was seen only at the two lower concentrations of 15 mM and 100 mM but not at 200 mM (Figure 1B), suggesting that the mmBCFA response may only be needed at lower concentrations of glucose. The stable isotope labeling patterns clearly reveal distinct responses to different concentrations of glucose, calling for further investigation of other fatty acid species.

The high concentrations of glucose revealed significant decreases in multiple species that are not compromised with lower glucose supplementation, including C18:0, C18:1n9, and C18:2n6. Specifically, C18:0 decreased from 8.8 ± 0.3% in control worms to 8.0 ± 0.1% in 100 mM and 7.7 ± 0.1% in 200 mM; C18:1n9 decreased from 15.0 ± 1.2% in control worms to 12.8 ± 0.6% in 100 mM and 12.8 ± 0.4% in 200 mM; and C18:2n6 decreased from 12.3 ± 0.9% in control worms to 10.2 ± 0.6% in 100 mM and 9.4 ± 0.6% in 200 mM (Figure 1B). The reduction in new C18:1n9 and C18:2n6 suggests that these fatty acid species are not being produced at adequate levels or that any newly synthesized molecules are specifically funneled to maintain a highly polyunsaturated species production. Here, the fatty acids were detected using an electron impact (EI) source for mass spectrometry, and, therefore, the only other PUFA that had sufficient detectable parent ions for analysis was C20:3n6. The amount of newly incorporated C20:3n6 did not change significantly from the controls, suggesting that C18:1n9 and C18:2n6 are consumed to funnel resources toward C20 PUFA populations (Figure 1B). 

To further support this funneling hypothesis, the total relative abundance of the fatty acid species involved in C20 PUFA production was quantified using GC-MS. The fatty acid profiles confirmed an increase in palmitate (C16:0) as previously seen at all concentrations, with this increase being the only significant change seen in the 15 mM treated animals in the species measured (Figure 1C). There was a similar increase in C16:0 at all concentrations, demonstrating that the increase in C16:0 does not continue to a rise in higher concentrations of glucose (Figure 1C). The C16:0 pool is elongated to C18:0, and the levels of C18:0 remain constant at all glucose concentrations, showing a specific impact on the accumulation of C16:0. C18:0 is converted to C18:1n9 by the FAT-7 desaturase, which was implicated in the response to glucose previously [7,8]. Here, there is a significant reduction in overall C18:1n9 levels in only the higher +gluc plates (100 mM and 200 mM). Because the precursors to C18:1n9 are elevated or maintained, we suspected that C18:1n9 was being consumed to produce the C20 PUFAs needed in the nematode. In fact, there was maintenance in the abundance of all measured PUFAs except for C18:2n6, where we found a trend of reduced levels in high +gluc plates, but significance was reached only on 100 mM plates (Figure 1C). Taken together, the stable isotope and GC-MS analysis show no significant changes in the C20 PUFA population at any glucose concentration, suggesting that new dietary carbon is funneled to maintain these populations. 

Elevated glucose in the diet is associated with decreased longevity, and therefore, we sought to test whether increasing glucose concentrations could lead to further impacts on lifespan [17]. For each glucose condition, the mean lifespan of the nematode was significantly reduced by approximately 30% (Figure 1D). There was no further lifespan reduction as the glucose concentration rose to 100 mM and 200 mM glucose. This is consistent with the fatty acid abundance and dynamics data, where we saw an impact at lower concentrations but not a correlation with glucose concentration for most fatty acid species. We hypothesize that the metabolic rewiring that occurs at 15 mM is largely sufficient to accommodate the increased glucose levels, but this observation requires a more careful probing of fatty acid dynamics.

### 3.2. A Recovery Period Is Needed to Drive the Shift in C18:1n9 Abundance

**Figure 2:**
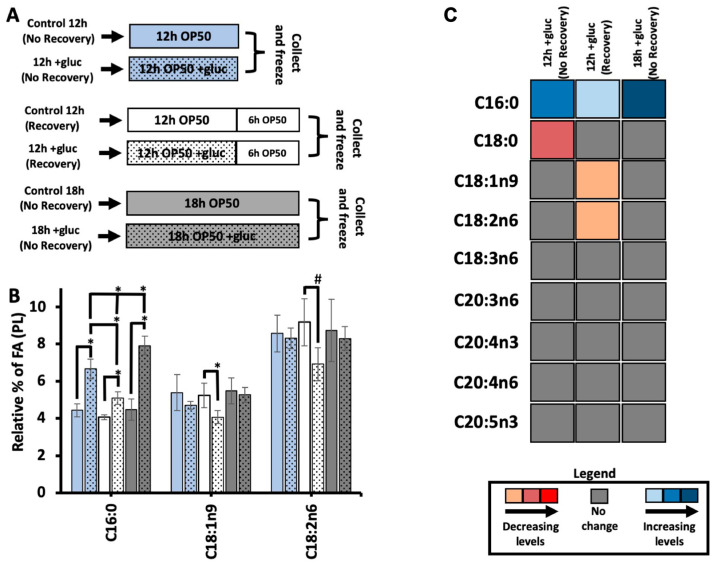
Recovery from glucose is required to observe the shift in C18:1n9. (**A**) For each comparison, the addition of glucose is represented by the inclusion of black dots vs. the control with no dots. The blue bars represent nematodes collected immediately after 12 h of stress (12 h; no recovery); the white bars are nematodes allowed to recover for 6 h in agarose plates seeded with 0.15 mg/mL of OP50 (12 h: recovery), and the gray bars are nematodes collected immediately after 18 h of stress (18 h; no recovery). (**B**) C16:0 SFA showed a significant increase in all glucose-stressed animals relative to its controls, and nematodes stressed for a longer period (18 h) showed a significantly larger accumulation of C16:0 compared to all other two-stress conditions. Both C18:1n9 and C18:2n6 maintained stable levels in (no recovery) animals but showed a significant decrease in +gluc 12 h (recovery) compared to their respective control. C18:1n9 decreased from 5.2% ± 0.6 in the control 12 h (recovery) to 4% ± 0.3 in +gluc 12 h (recovery), and C18:2n6 decreased from 9.1% ± 1.2 in the control 12 h (recovery) to 6.9% ± 0.8 in +gluc 12 h (recovery). (**C**) A heat map shows the alteration in FA levels comparing stressed animals to controls (+gluc/controls). Significant decreases are shown in light orange (little), dark orange (intermediate), and red (substantial). Significant increases are shown in light blue (little), median blue (intermediate), and dark blue (substantial). Gray bars indicate FAs that did not have a significant alteration. For all GC-MS analyses, values represent the mean ± SEM of at least 9 replicates. For statistical significance, *p* < 0.05 is indicated by *, and *p* < 0.1 is indicated by #, which was calculated using unpaired *t*-tests and F-tests to compare variances.

The higher concentrations of glucose revealed altered oleic acid dynamics consistent with the role of the FAT-7 desaturase in surviving glucose stress. Therefore, we further probed the kinetics of fatty acid metabolism with a focus on C18:1n9. First, the stable isotope labeling technique used here introduced ^13^C-OP50 on agarose media, which is free of nutrients to prevent the labeled bacteria from incorporating ^12^C from the unlabeled nutrients in the plate. A consequence of this protocol is that the nematodes are given a “recovery period” of 6 h on the labeling plates when there is no glucose stress. Because it was previously shown that removing nematodes from short glucose stress allowed for the recovery of development in PAQR-2 mutants, we examined the impact of this recovery period on fatty acids in the membrane [9]. To undertake this, we used GC-MS to quantify the abundance of saturated and unsaturated fatty acids in nematodes stressed for 12 h and frozen immediately following the stress (+gluc 12 h—no recovery) and nematodes stressed for 12 h followed by 6 h of a “recovery period” (+gluc 12 h—recovery). We also included nematodes that were stressed for 18 h and frozen immediately after stress (+gluc 18 h—No Recovery), which allowed us to identify changes that occurred not due to recovery but the extra hours on the plates (Figure 2A). To induce glucose stress, we selected 100 mM of glucose, which caused the most significant alterations in membrane dynamics and composition. 

We first quantified the abundance of C16:0 in the three conditions and found a significant increase in all glucose treatment groups regardless of the timing and recovery period. The increase in C16:0 with the recovery period was significantly less than in either of the treatment groups without a recovery period (Figure 2A). This trend suggests that the input of C16:0 to the membrane is higher in glucose conditions and that a higher input is reduced once glucose is removed from the plate. The amount of C16:0 continues to significantly rise between 12 h and 18 h of glucose feeding, supporting the hypothesis that the input of C16:0 is linked to glucose supplementation (Figure 2A). Next, we analyzed the relative levels of C18:1n9 in the PL membrane with and without a recovery. If the nematodes were not given a recovery period, oleic acid levels were not significantly different in 12 h glucose-stressed animals versus the controls (Figure 2B). When given a recovery period, +gluc 12 h (Recovery) showed a significant decrease when compared to controls where the level of C18:1n9 decreased from 5.2% ± 0.6 to 4.0% ± 0.3 (Figure 2B). C18:1n9 is converted to C18:2n6 via FAT-2, and we quantified similar trends in this species with a change from 9.1% ± 1.2 to 6.9% ± 0.8 after the recovery period (Figure 2B). This reduction was not a result of a longer time period as these fatty acid species, after 18 h of glucose stress, had no change in their overall levels (Figure 2B). 

The reduction in both the abundance of C18:1n9 and C18:2n6 after a recovery might show an overall reduction in PUFA production or a specific focus on the production of C20 PUFAs. Therefore, we quantified the relative fatty acid abundances for all major species in the nematode and in the three glucose treatment groups versus their respective controls (Figure 2C and Figure S1A). We found that there were no significant modifications in the relative abundance of any C20 PUFAs in any stress conditions (Figure 2C). The levels of decrease and increase shown in Figure 2C are indicated based on the levels of “*p*”, where light blue and orange had *p* values between 0.05 and 0.005, salmon pink and middle blue had *p* values between 0.005 and 0.002, and dark blue and red had *p* values between 0.002 and 0.001. The fatty acids that had significant changes in our previous results remained consistent as follows: increased levels of C16:0 in all treatment groups, decreased C18:1n9 abundance in the 12 h plus recovery, and decreased C18:2n6 abundance in the 12 h plus recovery treatment group (Figure 2C). In addition, there was a small but significant decrease in C18:0 in the 12 h group without recovery. Taken together, our data suggest that the reduction in C18:1n9 and C18:2n6 occurs as these fatty acids are converted to C20 PUFAs in the period following glucose exposure. 

### 3.3. The Abundance of Oleate and Linoleate Stabilizes with Longer Glucose Exposure

**Figure 3:**
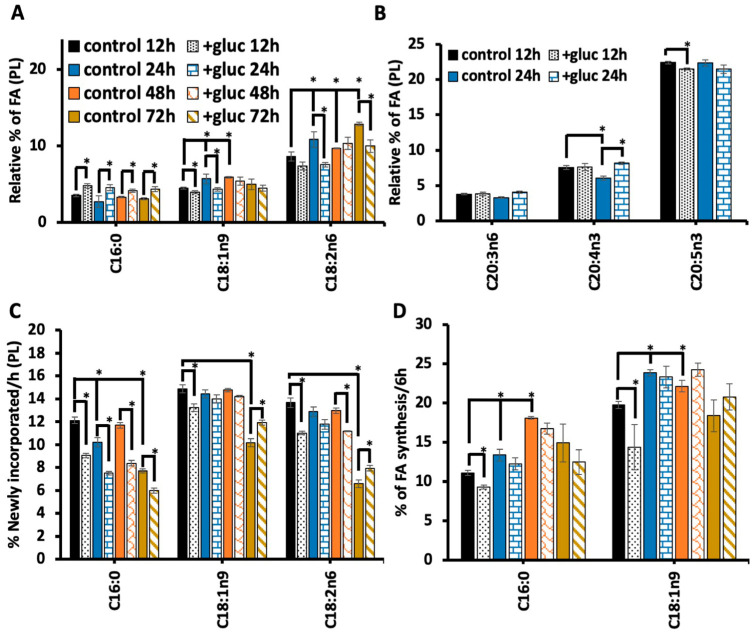
Longer stress periods stabilize oleic acid and linoleic acid levels. (**A**) The relative abundances of C16:0, C18:1n9 and C18:2n6 were found using GC-MS in the following treatment groups: +gluc 12 h (black dot), 24 h +gluc (blue horizontal brick), +gluc 48 h (orange hatch) and +gluc 72 h (gold diagonal stripes). The fatty acid abundances were compared to age-matched controls without glucose exposure: 12 h (black bars), 24 h (blue), 48 h (orange), and 72 h (gold). (**B**) The relative % of C20:3n6, C20:4n3 and C20:5n3 were assessed in 12 h and 24 h nematodes with and without glucose treatment. Small but significant differences were noted with a *. (**C**) The % of newly incorporated FA was quantified after incorporating dietary stable isotopes in the same treatment groups as defined above. (**D**) FA synthesis was calculated based on the abundance of isotopomers characteristic of synthesis (i.e., MW 272–284 for C16:0, and MW 301–313 for C18:1n9) after correcting for the natural abundance of isotopes and, correcting via a normalization factor, it accounted for differences in the distribution of isotopes in the diet. For all GC-MS analyses, values are the mean ± SEM of at least 4 replicates. Statistical significance (*p* < 0.05) is shown by * and was calculated using unpaired *t*-tests and F-tests to compare variances.

Once we found that the recovery period was essential to observe the alterations in C18:1n9 metabolism, we next tested the impact of longer durations of glucose stress. Nematodes were subjected to 100 mM glucose for 12 h, 24 h, 48 h, and 72 h, and all treatment groups had a 6 h recovery period to elicit the reduction in C18:1n9 and in C18:2n6 as well as to allow for stable isotope labeling. First, C16:0 abundance was considered, and with all durations of glucose stress, there was a significant increase in C16:0 (Figure 3A). Interestingly, the longer periods of glucose stress did not lead to further increases in C16:0 abundance. Because we saw an increase in C16:0 for 18 h compared to 12 h, we believe that the recovery period, along with metabolic rewiring, can compensate for the longer exposure to glucose. 

We next quantified the abundance of C18:1n9 with different lengths of 100 mM exposure. Here, we found a decrease in C18:1n9 from 4.4% ± 0.1 in the control 12 h to 3.9 ± 0.1 in +gluc 12 h and a greater decrease from 5.6% ± 0.6 in the control 24 h to 4.3 ± 0.2 in +gluc 24 h. However, the 48 h and the 72 h treatments did not lead to significant changes in C18:1n9 (Figure 3A). To interpret these data, we considered the oleic acid populations in the control populations, which revealed that the baseline C18:1n9 levels increased in 24 h and the 48 h controls (Figure 3A). The longer glucose exposure dictates that the lipid populations were examined in older animals, and although 24 h is a brief period, the first three days of adulthood are the peak reproductive period within the nematodes and are associated with glucose-independent metabolic changes [22,23]. Despite the impact of aging, these data are consistent with the upregulation of FAT-7, which requires time to stabilize the higher levels of the enzyme. 

To further understand the impact of the altered C18:1n9 levels on fatty acid elongation and the desaturation pathway, we next examined C18:2n6, and the immediate product of C18:1n9 desaturation (Figure 1A). The trends in C18:2n6 were similar to C18:1n9, with significantly decreased abundance from 10.8 ± 1.0% in the control for 24 h to 7.5 ± 0.3 in +gluc for 24 h (Figure 3A). Like C18:1n9, there was no significant change with 48 h of glucose feeding. However, the 72 h analysis revealed significantly higher levels of C18:2n6 in control animals compared to the young 12 h stressed controls and a significant decrease in C18:2n6 with 72 h glucose exposure (Figure 3A). Next, we examined if the reduction in C18:2n6 and C18:1n9 affected the abundance of the C20 PUFAs downstream. To perform this, we examined the levels of the major C20 PUFAs with 24 h glucose stress as that treatment had the greatest impact on the precursor populations (see Supplementary Figure S1B for all treatment durations). For C20:3n6, C20:4n3, and C20:5n3, there was some small but significant change in these pools; however, the extent of these changes was relatively minor. In fact, the C20:4n3 levels increased following 24 h of glucose (Figure 3B), supporting the hypothesis that the C18:1n9 produced by FAT-7 upregulation is funneled to preserve C20 PUFAs (Figure 3B). 

In addition to monitoring the overall abundance of these fatty acid pools, we implemented a stable isotope-labeling strategy to determine the flux in these populations. Consistent with the 12 h data, the amount of newly incorporated or isotopically labeled C16:0 was reduced with all durations of 100 mM glucose exposure (Figure 3C). In both C18:1n9 and C18:2n6, there was a significant reduction in the number of 13C fatty acids at 12 h, as seen previously. There were no significant changes in the labeling of C18:1n9 at 24 or 48 h despite reducing C18:1n9 abundance at 24 h. These data suggest that FAT-7 levels reach their peak within 24 h, and a turnover of 48 h is required for the fatty acid pool to stabilize. A similar trend is seen with C18:2n6, but there are significant differences at 48 h after labeling. We hypothesize that the upregulation of FAT-3, the enzyme that drives the conversion between C18:2n6 and C18:3n6, has slower kinetics. 

Finally, each of the fatty acid pools examined experienced a dramatic reduction in labeling after 72 h in the control nematodes. This reduction likely indicates an alteration in phospholipid metabolism as the animals leave the reproductive period, but it also uncovers the relative production of these fatty acids. New C16:0 production continues to be reduced (Figure 3C), which is consistent with the fact that the overall levels of C16:0 were still elevated at 72 h (Figure 3A). Both C18:1n9 and C18:2n6 have significant increases in new fatty acids compared to the unstressed controls (Figure 3C). The increases in these populations likely reflect the stabilization or activation of FAT-7 and FAT-3. 

The changes in fatty acid abundance can ultimately reflect increased production, increased dietary absorption, or decreased consumption. The stable isotope strategy we used here allowed us to define the origin of the oleate pool as altered with glucose supplementation. Under basal conditions, 11 ± 0.3% C18:1n9 was derived from de novo fatty acid synthesis consistent with past reports [11]. The contribution of synthesis to C18:1n9 production falls to 9.2 ± 0.2% in +gluc for 12 h (Figure 3D). The amount of synthesis is not statistically different in any of the longer treatment periods; however, it is interesting to note that the amount of synthesized fatty acids increases after 24 h and 48 h compared to 12 h in the controls (Figure 3D). The trends in synthesized C18:1n9 closely mirror the overall amount of stable isotope incorporation, suggesting that the processing of synthesized fatty acids is the driver of these dynamics. This is consistent with the composition of the bacterial diet, which has few overall changes in fatty acids [7]. 

Because C16:0 is the main product of de novo fatty acid synthesis, we quantified the amount of synthesized palmitate in the time course. Similar to C18:1n9, there was a decrease in synthesized C16:0 after 12 h and no significant changes at other time points (Figure 3D). We believe that this quantification shows that synthesis is initially reduced to prevent the further production of C16:0. This also shows that the elevated levels of C16:0 are not derived from using excess glucose to drive fatty acid synthesis in the nematode. Furthermore, there is an increase in synthesized C16:0 in 24 h and 48 h populations, reinforcing the altered fatty acid metabolism over the first few days of adulthood. Unlike C18:1n9, the pattern of synthesized C16:0 does not match overall labeling, implicating fatty acid absorption as a key contributor to palmitate metabolism. 

### 3.4. Living Bacteria Is Needed for the Impact of Glucose Stress on the Membrane

**Figure 4:**
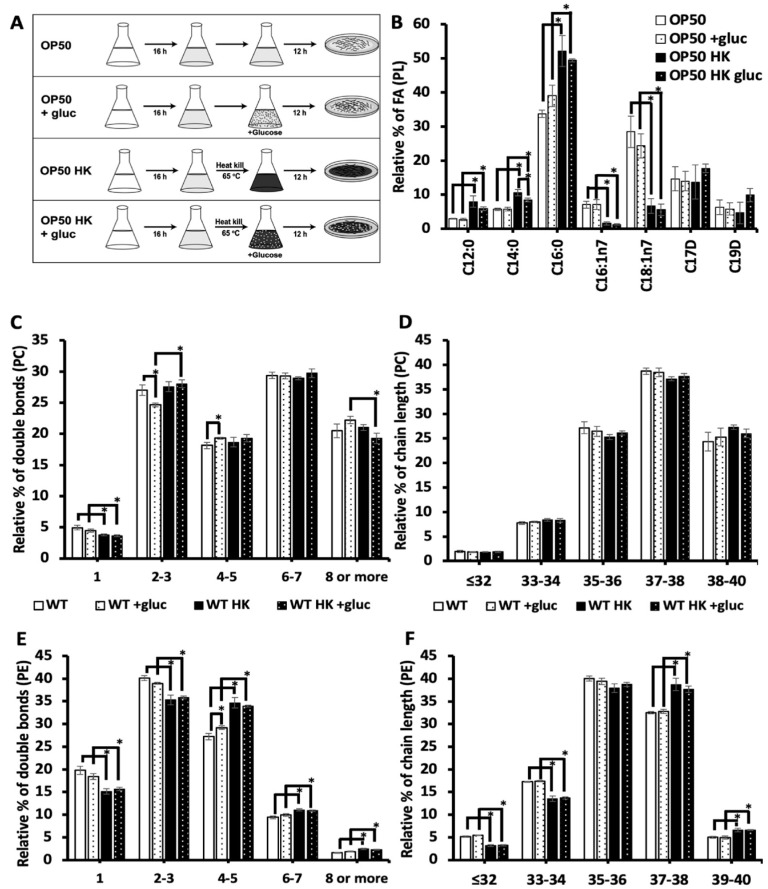
Glucose stress phenotypes require living bacteria. (**A**) Representation of the experimental design to test the impact of bacteria processing glucose. Nematodes were fed four different diets of living bacteria (OP50; white), living bacteria resuspended in glucose (OP50 +gluc; white with black dots), heat-killed bacteria (OP50 HK; black), and heat-killed bacteria were resuspended in glucose (OP50 HK +gluc; black with white dote). (**B**) The relative % of FA in bacteria was assessed using GC-MS and did not show statistical significance when comparing OP50 to OP50 +gluc and OP50 HK to OP50 HK +gluc. Comparing the killed bacteria to living bacteria showed a significant increase in all SFAs (C12:0, C14:0, and C16:0) and a significant decrease in all UFAs (unsaturated fatty acids) (C16:1n7 and C18:1n7). (**C**) The PCs in nematodes fed the different diets were evaluated using HPLC-MS/MS. A binned analysis of the number of double bonds in the PC populations is shown with statistically significant changes denoted. (**D**) There was no significant alteration in the relative % of the chain length of PC species in any condition. (**E**) PE species binned based on the double bond distribution (**E**) and chain length (**F**) are shown with statistically significant (*p* < 0.05) changes marked by an *.

The addition of glucose to the diet may induce excess saturated fatty acid production or alter membrane metabolism through another mechanism. For instance, it has been seen that glucose can cause oxidative stress through the increased production of advanced glycation agents [20]. Because the bacterial food source (OP50) is present during glucose exposure, bacteria could theoretically metabolize glucose and contribute to the effects of glucose stress. To test the impact of bacteria when processing glucose on the membrane composition, OP50 bacteria were grown in LB broth media, and then bacteria were separated into the following two groups: living and heat-killed bacteria (scheme shown in Figure 4A). Living bacteria were resuspended into fresh LB (OP50) for the control treatment and LB media containing 100 mM glucose (OP50 +gluc) for the stress treatment. In the other treatment group, bacteria were heat-killed at 65 °C for 20 min and resuspended into fresh LB media (OP50 HK (heat-killed)) and LB media containing 100 mM of glucose (OP50 HK + gluc).

To determine the impact of heat killing on the bacteria’s food source, we analyzed the fatty acid composition of the bacteria using GC-MS. Notably, we found that killing the bacteria significantly increased the level of saturated fatty acids, C12:0, C14:0, and C16:0, even in the absence of glucose (Figure 4B). Specifically, the level of C16:0, the most abundant saturated fatty acid in OP50, increased from 33.7 ± 1.0% for the dietary fatty acids to 52 ± 4.5% in heat-killed OP50. Additionally, there was a corresponding decrease in the level of unsaturated fatty acids, particularly C18:1n7, which decreased from 28.4 ± 4.5% in OP50 to 6.7 ± 2.1% in killed OP50. We next examined the impact of glucose in both populations and found that glucose did not affect the composition of living bacteria. There was a small but not significant increase in C16:0, which suggests that the accumulation of C16:0 in the nematodes is not a result of increased dietary C16:0. There was a small but significant decrease in C14:0 glucose in the killed OP50 with glucose, though the GC-MS analysis confirmed that the glucose did not have a major impact on the fatty acid composition of the killed bacterial populations (Figure 4B). 

To assess the impact of bacteria when processing glucose on the lipid composition of the nematodes, the phospholipid composition was analyzed using HPLC/MS-MS in animals fed living or killed bacteria. Here, we focused on the most abundant phospholipids of the membrane, phosphatidylcholine (PC) and phosphatidylethanolamine (PE) (see Supplementary Table S1 for full list). First, we considered the total number of double bonds present in the two fatty acid tails (i.e., 0–1, 2–3, 4–5, 6–7, and 8 or more) (Figure 4C) and the chain length (Figure 4D) of the PC populations. Feeding the animals with live OP50 +gluc led to a significant decrease in the level of species with 2–3 double bonds and an increase in species with 4–5 double bonds (Figure 4C). The decrease in lipid species with 2–3 double bonds is consistent with GC-MS data, as the fatty acids C18:1n9 and C18:2n6 are mostly present in this population. Animals that were fed a heat-killed OP50 +gluc did not experience any significant change in their degree of unsaturation, suggesting that living bacteria are needed for processing glucose. Except for a reduction in the populations with one double bond, there was no significant difference between the PC profiles in living versus killed bacteria without glucose treatment (Figure 4C). The phospholipid was also binned according to their chain length (the total amount of carbon present) as the length of phospholipids can influence the biophysical property of the membrane, such as thickness. However, there were no significant changes in the chain length of PC lipids when the worms were fed either living bacteria or heat-killed bacteria (Figure 4D). 

In analyzing the double bond distribution of the PE population, the animals were fed an OP50 + gluc diet and showed a significant increase in phospholipids with 4–5 double bonds (Figure 4E) and no changes in the distribution of chain lengths (Figure 4F). Again, there were no significant alterations in the level of unsaturation or chain length when the nematodes were fed an HK OP50 +gluc diet versus the killed diet without glucose supplementation (Figure 4E,F). Notably, there were significant changes in the chain length distribution of OP50 compared with OP50 HK, which made it difficult to disentangle the impact of the different dietary sources from the requirement for living bacteria to process the glucose. However, these results indicate that living bacteria may process glucose to drive the changes in the membrane lipid composition. 

## 4. Discussion

Here, we used increasing concentrations of glucose along with different treatment windows to map the metabolic responses to glucose stress in wildtype *C. elegans*. In performing this, we confirmed that a main feature of the glucose stress response is regulating the saturation balance in the PL membrane [8,9]. Although the regulation of membrane saturation is largely influenced by the activity of FAT-7, most of the data testing membrane composition used mild concentrations (15 to 20 mM) of glucose [7,9]. These concentrations were enough to show the accumulation of SFA in healthy nematodes, but species like C18:1n9 and C18:2n6 were maintained at stable levels [8]. At the higher concentrations used here, the abundance and the dynamics of C18:1n9 and C18:2n6 were compromised. However, there was no reduction in the fatty acids downstream of C18:2n6, indicating that the synthesis of C20 PUFA is prioritized. Each double bond is responsible for creating “kinks” in the FA structure, disrupting the tight packing of more linear molecules such as saturated fat. Previous fluorescence studies in cell culture have shown that the incorporation of fatty acids with four or more double bonds, such as C20:4n6, C20:5n3, and C22:6n3, increases membrane fluidity more than MUFAs [24]. We hypothesize that the decreased levels in all precursor FAs and the strict maintenance of PUFAs is a funneling process toward the maintenance of highly unsaturated fat to control membrane fluidity. 

Higher concentrations of glucose have been used to stress nematodes and evaluate the role of glucose in their health span, stress response, and lifespan. In fact, here, we defined 100 mM and 200 mM as high glucose conditions, but other studies used even higher concentrations [25,26]. However, it is important to consider that the concentrations here are supraphysiological and may not be reasonable models of glucose stress. The utility of these higher concentrations is to tax the nematode’s glucose response network, including PAQR-2, to reveal the changes in dynamics that may occur at lower concentrations, particularly in certain cell types or in certain organelles. It is striking to see how little the FA and the PL composition change with substantial amounts of exogenous glucose. The limited alterations in the membrane highlight the flexibility of the metabolic pathways to process a variety of diets and to support the composition of the lipid pool regardless of the input in wildtype animals. 

Although we can map the response to elevated glucose in the diet, it is not clear how excess glucose is driving these changes. Here, we found that this response required living bacteria, as we did not see the same changes with heat-killed bacteria. The role of living bacteria may be ensuring that the glucose is taken up by the nematodes or in processing the glucose to a metabolite. Although others have hypothesized that bacteria convert glucose to SFA, we observed a very modest increase in C16:0 with our experimental conditions. Because we did see an impact on the dynamics and longevity of animals in our study, we believe that the extra synthesis of C16:0 by bacteria does not account for the entirety of the increased C16:0 in the nematode. Furthermore, we found that there was a decrease in de novo fatty acid synthesis within the worm. Taken together, we hypothesized that elevated levels of glucose stress may have an added component. Several groups associated glucose accumulation with the increase in oxidative stress, ultimately affecting cell migration and promoting inflammation in human tissues [27,28,29,30,31]. In the membrane, the FAs formed a major oxidation target, resulting in the production of peroxidation products [32]. This occurs because the double bonds are particularly susceptible to the attack of reactive oxygen species, allowing for hydrogen abstraction from the carbon atoms [14]. In our experiments, we substantially increased glucose concentration, which could increase the levels of oxidative damage in the PUFAs. Thus, it is possible that the maintenance of PUFAs is an effort to correctly replace damaged lipids, avoiding membrane dysregulation and, consequently, apoptosis. In support of this model, the decrease in the lifespan seen in nematodes exposed to living bacteria fed glucose does not occur in dead bacteria supplemented with glucose [26].

To better understand the importance of C18:1n9 and C18:2n6 in the overall response to elevated levels of glucose, we proposed to test the effectiveness of recovery mechanisms by restoring membrane composition after its removal from the stress source. It was previously shown that removing PAQR-2 mutants from glucose stress allows for development to adulthood if the stress does not last longer than 12 h [9]. However, little is known about the influence of these mechanisms on the membrane’s adaptation to recovery. In our experiments, the labeling techniques give the nematodes a 6 h recovery period after stress, which creates an opportunity to further understand the process of membrane recovery. When no recovery period is present, C16:0 continues to accumulate, suggesting an increased input of SFA to the animal that overwhelms the fatty acid elongation and desaturation pathway. When glucose is removed, the C16:0 input is reduced, allowing the downstream enzymes to catch up in processing these fatty acids, and the decrease seen in C18:1n9 and C18:2n6 appears. We hypothesize that this reduced processing may be a result of an overall slower membrane turnover when there is an imbalance of SFA:UFA in the animal. 

## 5. Conclusions

In conclusion, our findings indicate that the regulation of oleic and linoleic fatty acids is essential to respond against high concentrations of glucose, which is consistent with the key role of FAT-7 in this response. In wildtype nematodes, the upregulation of fatty acid processing enzymes, likely through PAQR-2, eventually eliminates the lag in oleic acid processing. We believe that this study highlights the utility of stable isotope labeling in *C. elegans.* In fact, the labeling time course also revealed altered fatty acid synthesis and turnover as the control nematodes with no glucose exposure went through the first four days of adulthood. This observation shows the need for stable isotope studies over the entire *C. elegans* lifespan. 

## Data Availability

Data are contained within the article and Supplementary Materials.

## Supplementary Materials

The following supporting information can be downloaded at: https://www.mdpi.com/article/10.3390/metabo13121185/s1, Figure S1: PUFA Levels for All Glucose Treatment Durations; Table S1: Phospholipidomic profile of *C. elegans* fed OP50 with or without 100 mM glucose.
