# Supplementary material for: Oleic Acid Metabolism in Response to Glucose in C. elegans
Source: Metabolites. 2023 Dec 6;13(12):1185. doi: 10.3390/metabo13121185 (PMC10744850; doi:10.3390/metabo13121185)
Supplement: Supplementary file 1 [file metabolites-13-01185-s001.zip › Figure S1.pdf]

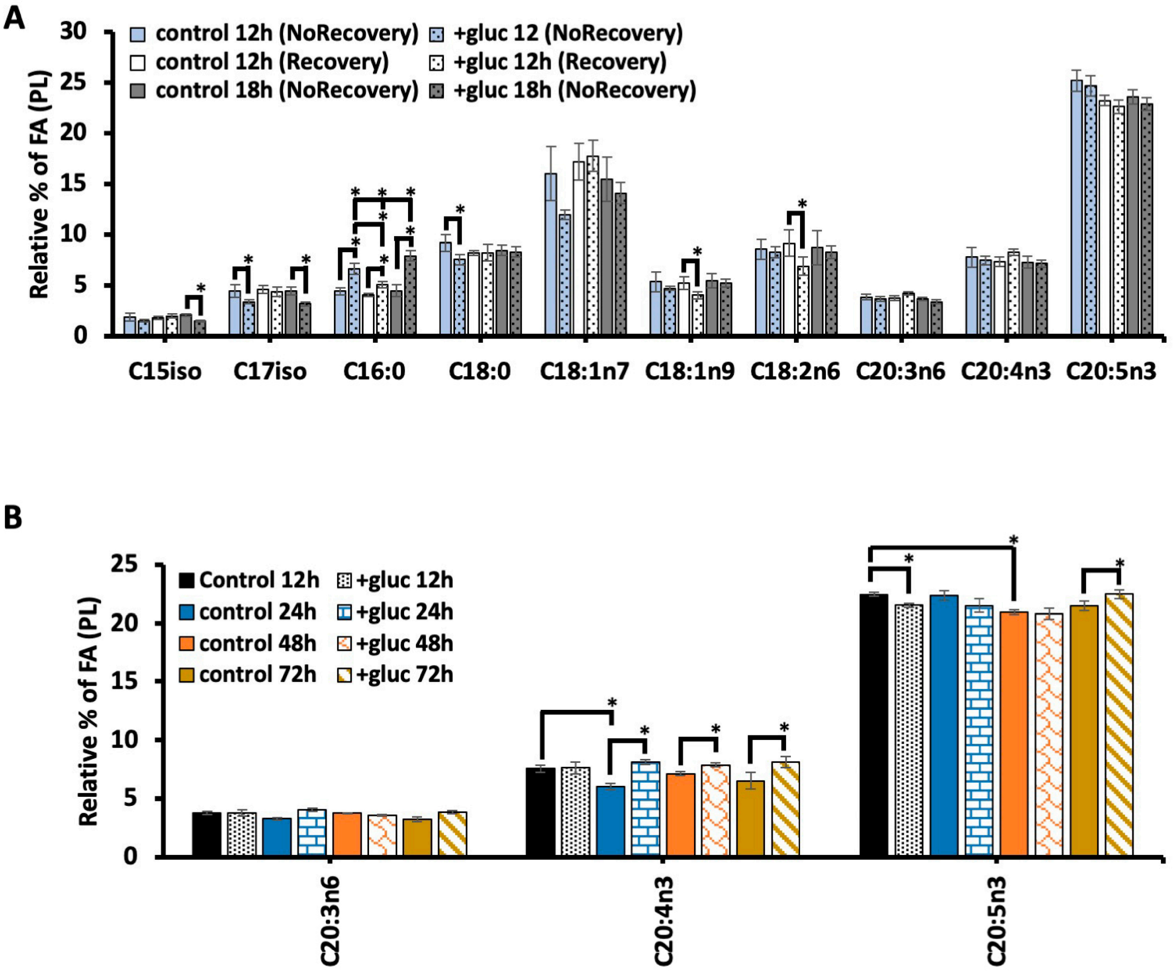

Figure S1: PUFA Levels for All Glucose Treatment Durations (**A**) Relative distribution for all the major FAs in the nematodes for recovery experiments. For each comparison, the addition of glucose is represented by the inclusion of black dots vs. the control with no dots. The blue bars represent nematodes collected immediately after 12 h of stress (12 h; no recovery); the white bars are nematodes allowed to recover for 6 h in agarose plates seeded with 0.15 g/mL of OP50 (12 h: recovery), and the gray bars are nematodes collected immediately after 18 h of stress (18 h; no recovery) (**B**) The relative distribution of the major PUFAs for longer duration of glucose stress for the following conditions: 12 h (black bars), +gluc 12 h (black dot), 24 h (blue), 24 h +gluc (blue horizontal brick), 48 h (orange), +gluc 48 h (orange hatch) and 72 h (gold) +gluc 72 h (gold diagonal stripes).
